# Cephalohematoma Infected With *Staphylococcus epidermidis* and Skull Abscess: A Case Report

**DOI:** 10.1002/ccr3.70703

**Published:** 2025-08-04

**Authors:** Saeedeh Parvaresh, Maedeh Jafari, Shahryar Eslami, Fatemeh Karami Robati, Mohammad Ali Jafari

**Affiliations:** ^1^ Department of Pediatrics School of Medicine, Kerman University of Medical Sciences Kerman Iran; ^2^ Clinical Research Development Unit Afzalipour Hospital, Kerman University of Medical Sciences Kerman Iran; ^3^ Department of Veterinary Basic Sciences Science and Research Branch, Islamic Azad University Tehran Iran

**Keywords:** case report, infections, skull, *Staphylococcus epidermidis*

## Abstract

Cephalohematoma is a self‐limiting lesion in 1%–2% of neonates. Cephalohematoma bleeds between the bone and periosteum and is limited to cranial sutures. Complications of the cephalohematoma include hyperbilirubinemia and secondary infection. We describe a neonate with cephalohematoma cultures positive for *S. epidermidis* as well as progressive deepening of the skull. There was no evidence of systemic infection, such as sepsis, meningitis, or osteomyelitis. In the ultrasound and brain MRI, the abscess was restricted to the scalp without involvement of the brain parenchyma and osteomyelitis of the bone. After a short period of intravenous antibiotics and drainage of the abscess, the patient was discharged from the hospital. To our knowledge, this is the first report of cephalohematoma infected with *Staphylococcus* *epidermidis* and skull abscess.

## 1 Introduction

Cephalohematoma in infants is caused by the accumulation of blood between the bones and the periosteum. This is secondary to rupture of the sentinel and diploic veins passing through the periosteum, leading to the accumulation of blood or serosanguineous fluid. The incidence rate is 0.4% to 2.5%, and for unknown reasons, cephalohematomas occur more often in male than in female infants [1, 2]. The most common site is the right parietal region. A cephalohematoma usually resolves over several weeks without complications. But it can become infected spontaneously through skin lesions or secondary to needle aspiration [3].

In this study, a case of cephalohematoma cultures positive for *S. epidermidis* as well as progressive deepening of the skull is presented.

## 2 Case History/ Examination

A 28‐day‐old boy was admitted to the hospital with occipital and purulent secretion of the scalp. The infant was delivered vaginally without local anesthesia, and instrumental assistance including forceps and vacuum was used. There was no manipulation of the newborn's scalp by the mother or any other person in the family. The occipital area was swollen at 15 days. Within 2 weeks, swelling increased in the occipital region. During the admission, the infant had no fever and no toxic appearance. In the examination of the cephalohematoma, 5 × 5 cm in the occipital, there was purulent discharge. Neurologic examination was normal. The size of the lymph nodes in the neck was also normal. Conversely, an initial assessment of blood tests was abnormal, and systemic infection was finalized. White cell count was 15.9 × 10^9^/L (neutrophils 82.2%, lymphocytes 17.8%). Hemoglobin level, platelet count, and C‐reactive protein were 10.7 g/dL, 312 × 10^9^/L, and 44 mg/L, respectively. Culture of the wound secretion was positive for *Staphylococcus* *epidermidis* . In the ultrasound of the skull, the structures of the brain were normal, and the echogenicity was increased in the right parietal region in favor of an abscess in the place of the previous cephalohematoma. Sepsis workup was done, including lumbar puncture. Cerebrospinal fluid examination was normal: Protein, 3.9 mg/L; glucose, and 50 mg/dL (45% of the random blood sugar value). The cells showed no inflammation, and the cerebrospinal fluid culture was negative.

## 3 Methods

**FIGURE 1:**
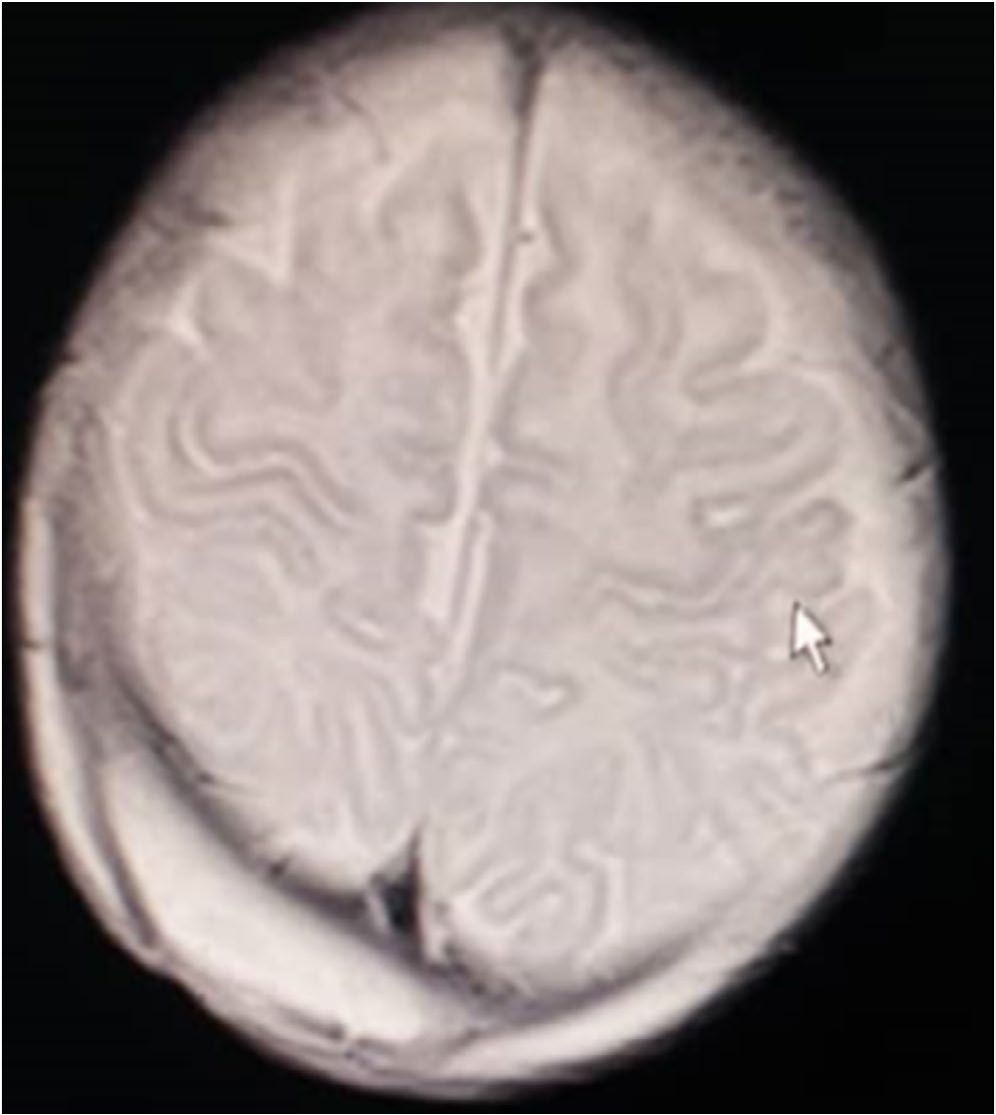
Brain MRI of head demonstrating a large collection in the right occipital region.

Blood cultures were negative. The purulent discharge was the hematoma of the *Staphylococcus* *epidermidis* . Vancomycin was started for the patient. An increase in the signal in the right peritoneal region was reported in the MRI that suggested an abscess. Brain abscesses are better seen with MRI than with a CT scan. There was no evidence of bone fracture or involvement of brain parenchyma or meningeal enhancement (Figure 1). Drainage of the abscess was done after 5 days, and antibiotic treatment was performed, and 50 cc of pus was removed.

## 4 Conclusion and Results

The infant received an intravenous antibiotic for 14 days and was discharged from the hospital after recovery. Follow‐up of the infant 1 month later showed a complete recovery.

## 5 Discussion

The cephalohematoma is usually resolved spontaneously and the possibility of infection is rare. In 1971, Lee reported 10 cephalohematoma infections [4]. Infection risk factors included electrode for fetal monitoring or trauma during delivery. Our patient did not have a risk factor for scalp infection. The source of infection was unknown in this patient. Culture of the wound secretion was positive with *Staphylococcus* *epidermidis* .

The cephalohematoma usually occurs in the first 2 weeks of life, like in our patient. Infection of cephalohematoma in the first 2 weeks of life is usually due to systemic infection, such as bacteremia and sepsis. After the first 2 weeks of life, the cause of infection is the spread of skin infection into deep scalp tissue [3, 5].

Infected cephalohematoma may be manifested by localized skin infection and epidural abscess or systemic disease associated with fever, septicemia, and meningitis [6]. In a study by Chery et al., the most common microbial agent of infectious cephalohematoma was *Escherichia coli* and *Staphylococcus aureus* [7]. Less common organisms included *Staphylococcus epidermidis* , Pseudomonas sp., Proteus sp., Bacteroides sp., and Salmonella sp. [8].

Clinical diagnosis of infectious cephalohematoma is difficult. Initial tests include blood culture, white blood cell count, and CRP. In addition, smear and culture from pus and cerebrospinal fluid are other options. The gold standard for diagnosis is the isolation of microorganisms in the culture of Needle Aspiration from pus. Ultrasonography is performed to find out the swelling and abscess in the soft scalp tissue. The goal of performing CT scan and MRI is to find the evidence of osteomyelitis and epidural abscess [9, 10]. CT scan shows abnormality and bone fractures, and if MRI is not available, the method is optional [11].

The MRI method for detecting osteomyelitis has a sensitivity of 95% [12]. Treatment of infectious cephalohematoma includes drainage and intravenous antibiotics against *Staphylococcus aureus* and gram‐negative bacilli such as E. coli. In our patient, we started the treatment with vancomycin. The duration of antibiotic therapy depends on the severity of the lesion and bone involvement as well as synchronous meningitis. The need for more treatment protocols is based on the patient's condition.

## Author Contributions

**Saeedeh Parvaresh:** conceptualization, project administration, supervision, validation, visualization. **Maedeh Jafari:** conceptualization, data curation, formal analysis, funding acquisition, investigation, methodology, project administration, resources, software, supervision, validation, visualization, writing – original draft, writing – review and editing. **Shahryar Eslami:** conceptualization, supervision. **Fatemeh Karami Robati:** writing – review and editing. **Mohammad Ali Jafari:** conceptualization.

## Ethics Statement

Informed consent received from all patients before starting the work and this study was approved by the Ethics Committee of Kerman University of Medical Sciences in Iran (Ethical Code: IR.KMU.AH.REC.1400.145). All clinical investigations were conducted according to the Declaration of Helsinki principles.

## Consent

Written informed consent was obtained from all of the patients.

## Conflicts of Interest

The authors declare no conflicts of interest.

## Data Availability

All data generated or analyzed during this study are included in this published article.
